# Right Ventricular Function and Its Coupling With Pulmonary Circulation in Precapillary Pulmonary Hypertension: A Three-Dimensional Echocardiographic Study

**DOI:** 10.3389/fcvm.2021.690606

**Published:** 2021-07-02

**Authors:** Yidan Li, Dichen Guo, Juanni Gong, Jianfeng Wang, Qiang Huang, Shu Yang, Xinyuan Zhang, Huimin Hu, Zhe Jiang, Yuanhua Yang, Xiuzhang Lu

**Affiliations:** ^1^Department of Echocardiography, Heart Center, Beijing Chao-Yang Hospital, Capital Medical University, Beijing, China; ^2^Department of Respiratory and Critical Care Medicine, Beijing Chao-Yang Hospital, Capital Medical University, Beijing, China; ^3^Department of Intervention, Beijing Institute of Respiratory Medicine, Beijing Chao-Yang Hospital, Capital Medical University, Beijing, China; ^4^Philips (China) Investment Co. Ltd., Beijing, China

**Keywords:** right ventricular-arterial coupling, pulmonary hypertension, right ventricular dysfunction, three-dimensional echocardiography, prognosis

## Abstract

**Objective:** To assess right ventricular (RV) function and RV-pulmonary arterial (PA) coupling by three-dimensions echocardiography and investigate the ability of RV-PA coupling to predict adverse clinical outcomes in patients with precapillary pulmonary hypertension (PH).

**Methods:** We retrospectively collected a longitudinal cohort of 203 consecutive precapillary PH patients. RV volume, RV ejection fraction (RVEF), and RV longitudinal strain (RVLS) were quantitatively determined offline by 3D echocardiography. RV-PA coupling parameters including the RVEF/PA systolic pressure (PASP) ratio, pulmonary arterial compliance (PAC), and total pulmonary resistance (TPR) were recorded.

**Results:** Over a median follow-up period of 20.9 months (interquartile range, 0.1–67.4 months), 87 (42.9%) of 203 patients experienced adverse clinical outcomes. With increasing World Health Organization functional class (WHO-FC), significant trends were observed in increasing RV volume, decreasing RVEF, and worsening RVLS. RV arterial coupling (RVAC) and PAC were lower and TPR was higher for WHO-FC III+IV than WHO-FC I or II. The RVEF/PASP ratio showed a significant correlation with RVLS. RVAC had a stronger correlation with the RVEF/PASP ratio than other indices. Multivariate Cox proportional-hazard analysis identified a lower 3D RVEF and worse RVLS as strong predictors of adverse clinical events. RVAC, TPR, and PAC had varying degrees of predictive value, with optimal cutoff values of 0.74, 11.64, and 1.18, respectively.

**Conclusions:** Precapillary-PH with RV-PA uncoupling as expressed by a RVEF/PASP ratio <0.44 was associated with adverse clinical outcomes. PAC decreased and TPR increased with increasing WHO-FC, with TPR showing better independent predictive value.

## Introduction

Right ventricular (RV) adaptation to chronic pulmonary hypertensive (PH) syndromes is a significant determinant of long-term outcomes. RV failure is prognostic of adverse outcomes in patients with PH, and RV function is the most important prognostic determinant of survival in various forms of PH (1–3).

RV adaptation to an increased PA load is a key determinant of outcomes in patients with precapillary PH (4). Furthermore, the RV is highly afterload sensitive, and it is generally accepted that RV-pulmonary arterial (PA) coupling in PH is a load-independent indicator of RV function that can powerfully detect RV dysfunction well before an obvious decrease in the RV ejection fraction (RVEF) is detected (5). Assessment of RV-PA coupling should be implemented in order to investigate the mechanisms of action of targeted therapies in PH, which decrease RV afterload regardless of the intrinsic myocardial effect, even if not considered the primary endpoint (6).

Right heart function and its coupling to pulmonary circulation play vital roles in the prognosis of PH. At present, pulmonary vascular resistance and compliance are two important indexes of PH in comprehensive clinical evaluation (7). PA compliance (PAC) strongly contributes to RV afterload, and clinical studies investigating changes in PAC compliance are needed (8). At present, the gold standard for measuring RV-PA coupling is multibeat RV pressure-volume assessment using high-fidelity catheters. Clinically accurate measurement of RV-PA coupling requires invasive measurements, and multiple-beat and single-beat methods are used for calculating effective arterial elastance (Ea) and RV end-systolic elastance (Ees) (9, 10). Right cardiac catheterization is still the “gold standard” for diagnosed pulmonary hypertension; however, due to technical complexity and considering the cost and risk of invasive surgery, non-invasive testing tools are necessary. Meanwhile, some studies have tried to use non-invasive echocardiographic evaluation methods. The ratio of tricuspid annular plane systolic excursion (TAPSE) to PA systolic pressure (PASP) is a non-invasive surrogate indicator of RV-PA coupling for clinical application (11, 12).

However, as we know, TAPSE, as only a one-dimensional parameter, cannot fully reflect the overall function of the RV. With the growing availability of three- dimensional (3D) imaging techniques such as magnetic resonance imaging (MRI) and 3D echocardiography (3DE), assessment of RV shape and functional changes has become more feasible. Three-dimensional modalities offer exceptional accuracy and reproducibility for the assessment of RV shape and function. With recent advances in 3DE, RV volumes measured by this technique can be used to evaluate RV-PA coupling. Specifically, 3DE can be used to measure RV shape and volume for the assessment of RV-PA coupling (13). Moreover, evaluation of the change in RV volume with 3DE may represent a novel approach for non-invasive assessment of RV-PA coupling. The objectives of the present study were to evaluate RV function and RV-PA coupling across patients of different World Health Organization functional class (WHO-FC) stages by quantitative 3DE analysis and to assess their predictive values for adverse clinical outcomes in precapillary PH patients.

## Methods

### Ethics Statement

This study was conducted according to the principles defined in the Declaration of Helsinki. The study protocol was approved by the Ethics Committee of Beijing Chao-yang Hospital. All participants in this study signed the written informed consent forms prior to the initiation of this study.

### Study Protocol

This was a single-center, retrospective longitudinal cohort study consisting of consecutive precapillary PH patients treated from February 2014 to July 2019. Consecutive patients aged ≥18 years with a diagnosis of precapillary PH were included. Precapillary PH was defined by mPAP >20 mmHg, PAWP ≤ 15 mmHg and PVR >3 WU at rest as assessed by RHC (14).

All clinical characteristics and laboratory data at baseline were obtained from standardized clinical data by PH physicians (Drs. Yang YH and Gong JN). Patients underwent echocardiography, 6-min walk test and N-terminal pro-B-type natriuretic peptide (NT-proBNP) measurement within a 48-h interval. The WHO-FC for each patient was determined.

**Figure 1:**
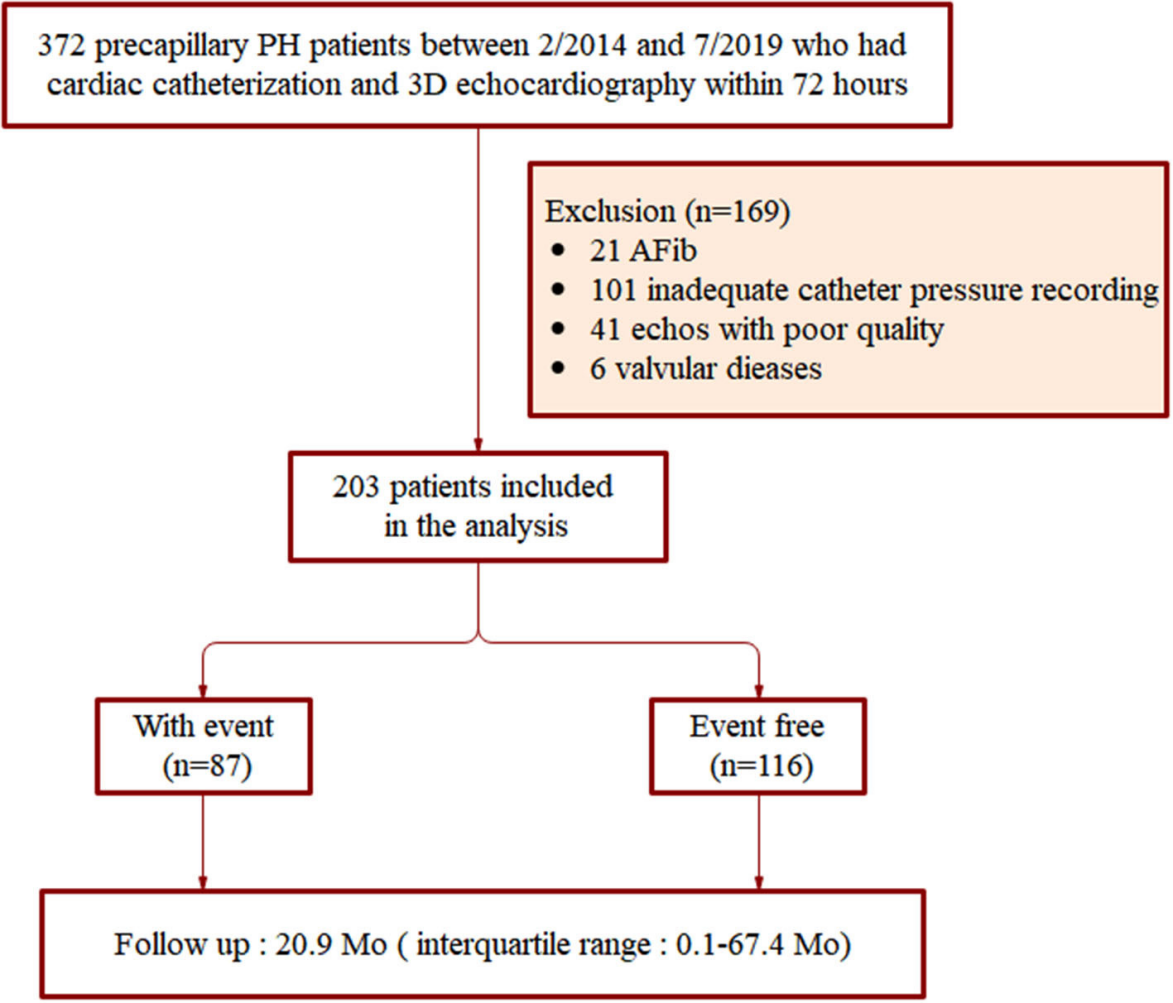
Flow chart of patient inclusion.

The demographic and clinical characteristics of all patients included in the study were obtained through interviews and through inspection of the hospital database. The exclusion criteria included: incomplete clinical and catheter pressure recording data, atrial fibrillation, moderate or severe mitral or aortic valvular disease, and echocardiography imagines of poor quality. Initially, a total of 372 precapillary PH patients were registered. Among these patients, 101 cases did not have complete clinical or RHC recording; 21 patients had atrial fibrillation; and 6 had tricuspid valve prolapse. Another 13 patients had severe dilation of the RV leading to incomplete display and resulting in poor quality echocardiography imagines, and in 28 patients, the endocardium of the free wall of the RV was unclear. After application of the exclusion criteria, a total of 203 patients were finally included in this study (Figure 1).

### 3DE

Images for the determination of RV volume were obtained with a Philips EPIQ 7C Doppler ultrasound machine (Philips Healthcare, MA, USA) equipped with an X5-1 transducer (1–5 MHz). The RV-focused apical four-chamber (4Ch) dataset was obtained for a single breath-hold, and the electrocardiogram was gated over a maximum of 6 beats. Throughout the cardiac cycle scan, the entire RV cavity was within the scan volume. The optimized imaging depth and sector width were required to obtain the highest frame rate. The RV volume and function quantitative analyses were performed offline using the new ML approach (3D Auto RV, Philips Healthcare) for data management. The resulting dynamic surface model could optionally be adjusted in end-diastole (ED) or end-systolic (ES). In the View Adjustment workflow step, the user could manually adjust the image landmarks. Accordingly, a RV-focused four-chamber view and a short-axis view were derived by the system. Also, the heart cycle to be analyzed could be changed in this state. According to the software guidelines, RV 3D dynamic surface models were generated, from which end-diastolic volume (EDV) and end-systolic volume (ESV) were obtained and stroke volume (SV) was calculated as: SV = EDV–ESV (Supplementary Figure 1) (15).

### RV Structural and Function Parameters

The following echocardiographic RV structural parameters were recorded: RV end-diastolic volume, RV end-diastolic volume indexed to body surface area (BSA), RV end-systolic volume, RV end-systolic volume indexed to BSA, RV basal diameter, RV mid-diameter, RV longitudinal diameter, RV basal diameter/LV basal diameter, and LV end-diastolic eccentricity index. Pericardial effusion was also be recorded.

RV function was evaluated based on the 2015 American Society of Echocardiography guidelines for cardiac chamber quantification by echocardiography in adults (16), including a RV index of myocardial performance (RIMP), RV free wall longitudinal strain (RVLS), tricuspid annular plane systolic excursion (TAPSE), RV fractional area change (FAC), and tissue Doppler-derived tricuspid lateral annular systolic velocity (S′). The RVLS, RV FAC, and TAPSE data were obtained from 3D echocardiographic RV datasets; RIMP and S′ were obtained by tissue Doppler methods. The 3D-based RVEF was calculated using the formula: RVEF = (EDV–ESV)/EDV.

### RV-PA Coupling

RV-PA coupling was assessed according to the Ees/Ea ratio. Ees represents RV contractility, and Ea represents RV afterload. RV-PA coupling is represented by Ees/Ea. RV Ees was determined as follows: Ees = RV ESP/ESVI (17). Ea was calculated as follows (18): Ea = RV ESP/SVI, where RV ESP is the RV end-systolic pressure, ESVI is the end-systolic volume index, and SVI is the stroke volume index. mPAP was used as a surrogate for RV ESP.

Pulmonary arterial compliance (PAC) was calculated as SV divided by PA pulse pressure (SV/systolic-diastolic PAP) (mL/mm Hg). Total pulmonary resistance (TPR) was calculated as mPAP/CO (18). SV was calculated as CO divided by heart rate.CO and SV were indexed for body surface area to obtain both the cardiac index (CI) and SVI. ESV was obtained by 3DE RV volume analysis, and CO and PA pressure were measured by RHC.

### RHC

A Swan-Ganz thermodilution catheter (Edwards Lifesciences, Irvine, CA, USA) was inserted into the right inferior PA by experienced physicians (Drs. Wang JF and Huang Q). The following parameters were measured under stable hemodynamics: RA pressure, mPAP, pulmonary vascular resistance, and CI. Pulmonary vascular resistance (PVR) in Wood units (WU) was calculated using the equation: PVR = (mPAP – PAWP)/CO. Echocardiography and RHC were performed with an interval of <72 h for all patients.

### Clinical Outcomes

Clinical outcomes were analyzed in all precapillary PH patients according to pre-defined adverse clinical events. Adverse clinical events were defined as: (i) PH-related hospitalization with RV function deterioration, (ii) deterioration of PH requires further interventional or surgical treatment, including pulmonary endarterectomy and/or balloon pulmonary angioplasty, or (iii) death. The cause of death or hospitalization was adjudicated by the authors (Drs. Yang YH and Gong JN), after a review of the relevant medical history and related documentation. All patients were followed up until occurrence of clinical events or the end of the study period.

### Statistical Analysis

All statistical analyses were conducted using SPSS version 23 (SPSS Software, Chicago, IL, USA) and MedCalc 16.1 (MedCalc Software, Mariakerke, Belgium). The one-sample Kolmogorov-Smirnov test was used to determine the normal distribution of all data. The continuous variables are presented as mean ± standard deviation (SD), while categorical variables are presented as absolute numbers and percentages. The independent sample *t*-test was used to identify significant between-group differences in normally distributed data. The relationships between RVEF and other clinical and echocardiographic variables were assessed using Pearson's or Spearman's correlation coefficients. Continuous variables were compared across the three cohorts using one-way analysis of variance (ANOVA) for quantitative variables and Chi square tests or exact Fisher Tests for categorical variables.

The associations between variables and the combined clinical end point were tested using univariate Cox proportional-hazards analysis, with results presented as hazard ratios (HRs) and two-sided 95% confidence intervals (CIs). Multivariate survival analysis included all variables with a *P* < 0.10 on the univariate analysis as well as previously described prognostic parameters. Survival receiver operating characteristic (ROC) curves were constructed for the assessment of the sensitivity and specificity of the used predictors for the prediction of adverse clinical events. The optimal cut-off values for predicting survival were calculated using Youden's index method. Kaplan–Meier analysis was implemented, dividing patients according to RV function and RV-PA coupling indexes.

To assess the magnitude of the variability caused by the manual corrections, an intra- and inter-reader variability analysis was conducted. Variability was expressed in terms of coefficients of variation (CoVs), calculated as the absolute difference between the corresponding pairs of repeated measurements as a percentage of their mean, and intraclass correlation coefficients (ICCs). Statistical significance was defined as a two-sided *P* < 0.05.

## Results

### Characteristics of the Patient Population

A total of 203 patients (28.1% males; mean age, 49.2 ± 14.5 years) with precapillary PH were enrolled and divided into three groups according to WHO-FC (Table 1). The WHO-FC I, II, and III/IV groups included 20 (9.9%) patients, 130 (64.0%) patients, and 53 (26.1%) patients, respectively. Across the WHO-FC groups, significant trends were observed for increasing NT-proBNP, right atrium pressure (RAP), mPAP, and PVR and decreasing 6-min walk distance (6MWD) with a decrease in CI. Of 203 enrolled subjects, 87 (42.9%) experienced adverse clinical outcomes during the follow-up period. Overall, 3 patients died, 43 underwent pulmonary endarterectomy and/or balloon pulmonary angioplasty, and 41 were hospitalized for PAH.

**Table 1:** Demographic and baseline clinical characteristics of patients with precapillary PH.

**Variable**	**Study cohort** ** (*N* = 203)**	**WHO-FC I** ** (*n* = 20)**	**WHO-FC II** ** (*n* = 130)**	**WHO-FC III + IV** ** (*n* = 53)**	**Overall** ***P*-value**
Age (y)	49.2 ± 14.5	45.2 ± 15.0	49.8 ± 14.3	49.1 ± 14.8	0.413
Male, *n* (%)	57 (28.1)	5 (25)	41 (31.5)	11 (20.8)	0.324
BSA (m^2^)	1.7 ± 0.2	1.7 ± 0.2	1.7 ± 0.2	1.7 ± 0.1	0.501
6MWD (m)	378 (98–630)	450 (249–630)	386 (98–610)	331 (190–500)	** <0.001**
NT-proBNP (pg/ml)	1230.8 ± 1436.5	659.2 ± 1066.3	1066.1 ± 1779.9	1899.8 ± 1744.9	**0.004**
Etiologies					0.233
CTEPH	95 (46.8)	7 (35.0)	62 (47.7)	26 (49.1)	
Connective tissue disease	44 (21.6)	4 (20.0)	29 (22.3)	11 (20.8)	
Idiopathic	37 (18.2)	3 (5.0)	21 (16.2)	13 (24.5)	
Congenital heart disease	17 (8.4)	3 (15.0)	12 (9.2)	2 (3.8)	
Heritable	5 (2.5)	1 (5.0)	3 (2.3)	1 (1.8)	
Drug use	5 (2.5)	2 (10.0)	3 (2.3)	0 (0.0)	
**Basic hemodynamic data**					
RAP (mm Hg)	5.6 ± 4.5	3.7 ± 3.0	5.4 ± 4.2	6.8 ± 5.3	**0.033**
mPAP (mm Hg)	47.9 ± 13.5	41.5 ± 17.8	47.6 ± 12.4	51.2 ± 13.7	**0.021**
CI (L/min per m^2^)	2.4 ± 0.7	2.9 ± 0.5	2.5 ± 0.7	1.9 ± 0.5	** <0.001**
PVR (Wood units)	11.0 ± 6.2	6.9 ± 3.9	10.1 ± 5.3	14.9 ± 7.1	** <0.001**
PH medical treatment					0.147
PDE-5 inhibitors	96 (47.3)	9 (45.0)	61 (46.9)	26 (49.1)	
Endothelin receptor antagonists	75 (36.9)	7 (35.0)	46 (35.4)	22 (41.5)	
Combined therapy	126 (62.1)	4 (20.0)	79 (60.8)	43 (81.1)	
Follow-up (mo)	24.5 (0.1–67.7)	27.5 (0.1–60.3)	24.4 (0.3–67.4)	23.5 (0.3–67.7)	0.704
**Adverse clinical events**					
PH-related death	3 (1.5)				
PH-related hospitalization	41 (20.2)				
Balloon pulmonary angioplasty	36 (17.7)				
Pulmonary endarterectomy	7 (3.4)				

*BSA, body surface area; 6MWD, 6-min walk distance; NT-proBNP, N-terminal pro-B-natriuretic peptide; RA, right atrium; RAP, RA pressure; mPAP, mean PA pressure; CI, cardiac index; PVR, pulmonary vascular resistance. Data are expressed as mean ± SD, number (percentage), or median (interquartile range). P-values indicate statistical significance between groups. Bold values indicate statistical significance of a difference between the tested parameters*.

### RV Size and Function Across WHO-FC Groups

RV dilatation was found to be higher in patients with precapillary PH with worsening WHO-FC. The results in Table 2 show that patients with WHO-FC III+IV had larger RV volumes, with EDV (*P* = 0.016 and *P* < 0.001, respectively) and ESV (*P* = 0.006 and *P* < 0.001, respectively) values significantly greater than those in patients in the WHO-FC I and II groups. A higher RV volume and RV basal and mid-diameters with worse RV systolic function accounted for RV afterload, while no statistical difference in RV longitudinal diameter was observed (*P* = 0.949). RV functional parameters showed progressive deterioration of the RV with worsening WHO-FC. RVEF, TAPSE, RV FAC, and S′ were lower and RVLS was worse in the WHO-FC III+IV group than in the WHO-FC I and II groups. Across the WHO-FC stages, significant trends were observed for higher RV volume, lower RVEF, and worsened RVLS.

**Table 2:** Comparison of RV structural parameters and functional indices and RV coupling to pulmonary circulation among groups with differing WHO-FC.

**Variable**	**Study cohort** ** (*N* = 203)**	**WHO-FC I** ** (*n* = 20)**	**WHO-FC II** ** (*n* = 130)**	**WHO-FC III + IV** ** (*n* = 53)**	**Overall** ***P***
**RV structural parameters**					
RV EDV (ml)	114.4 ± 38.9	90.9 ± 41.5^*^	112.8 ± 34.9	127.1 ± 42.7	**0.001**
RV EDVI (ml/m^2^)	67.6 ± 22.1	53.2 ± 23.7^*^	66.3 ± 19.4	76.1 ± 24.5	** <0.001**
RV ESV (ml)	72.8 ± 31.2	50.3 ± 27.4^*^^§^	69.8 ± 27.0	88.7 ± 34.9	** <0.001**
RV ESVI (ml/m^2^)	43.0 ± 17.8	29.4 ± 15.5^*^^§^	41.0 ± 15.3	52.9 ± 19.6	** <0.001**
RVD1 (mm)	46.0 ± 6.8	44.1 ± 6.8^‡^	45.2 ± 6.5	48.5 ± 6.8	**0.004**
RVD2 (mm)	42.1 ± 7.8	38.3 ± 7.3^†^	41.2 ± 6.9	45.9 ± 9.0	**0.001**
RVD3 (mm)	74.7 ± 8.1	74.1 ± 8.8	74.7 ± 7.9	74.9 ± 8.2	0.949
RV/LV	1.4 ± 0.3	1.2 ± 0.2^*^	1.3 ± 0.3	1.5 ± 0.3	** <0.001**
EI	1.4 ± 0.3	1.2 ± 0.1^†^	1.3 ± 0.2	1.5 ± 0.3	**0.002**
**RV function parameters**					
RVEF (%)	37.5 ± 9.1	45.6 ± 8.9^*^^§^	38.9 ± 8.7	31.2 ± 5.5	** <0.001**
RVLS (%)	−18.6 ± 5.5	−22.2 ± 4.5^*^	−19.8 ± 5.1	−14.2 ± 4.5	** <0.001**
TAPSE (mm)	15.5 ± 5.2	18.5 ± 4.9^*^	16.5 ± 5.0	12.1 ± 4.1	** <0.001**
RV FAC (%)	28.4 ± 8.8	36.1 ± 8.0^*^^§^	29.6 ± 8.4	23.1 ± 7.1	** <0.001**
RIMP	0.7 ± 0.3	0.6 ± 0.1^*^	0.7 ± 0.2	0.9 ± 0.3	** <0.001**
S′ (cm/s)	10.8 ± 3.1	12.6 ± 3.3^*^	11.1 ± 3.2	9.4 ± 2.3	** <0.001**
**RV-PA coupling**					
RVEF/SPAP	0.49 ± 0.22	0.63 ± 0.26^*^	0.52 ± 0.22	0.36 ± 0.13	** <0.001**
RVAC	0.87 ± 0.66	1.74 ± 1.23^*^^§^	0.88 ± 0.52	0.50 ± 0.23	** <0.001**
TPR (mm Hg/ml)	13.57 ± 6.97	9.50 ± 7.39^*^	12.46 ± 5.52	17.80 ± 8.10	** <0.001**
PAC (ml/mm Hg)	1.06 ± 0.57	1.56 ± 0.50^*^^§^	1.10 ± 0.58	0.83 ± 0.30	** <0.001**

*RVEF, RV ejection fraction; RVLS, RV longitudinal strain; FAC, fractional area change; RIMP, RV index of myocardial performance; TAPSE, tricuspid annular plane systolic excursion; RV EDV, RV end-diastolic volume; RVEDVI, RV end-diastolic volume indexed to BSA; RVESV, RV end-systolic volume; RVESVI, RV end-systolic volume indexed to BSA; RVD1, RV basal diameter; RVD2, RV mid-diameter; RVD3, RV longitudinal diameter; RV/LV, RV basal diameter/LV basal diameter; EI, LV end-diastolic eccentricity index; PE, Pericardial effusion; BSA, body surface area; LV, left ventricular. Data are expressed as mean ± SD. P-values indicate statistical significance between groups. Bold values indicate statistical significance of a difference between the tested parameters*.

* *P <0.001 from WHO III+IV*.

† *P <0.01 from WHO III+IV*.

‡ *P <0.05 from WHO III+IV*.

§ *P <0.01 from WHO II*.

### RV-PA Coupling Indices and Relationships in Precapillary PH

**Figure 2:**
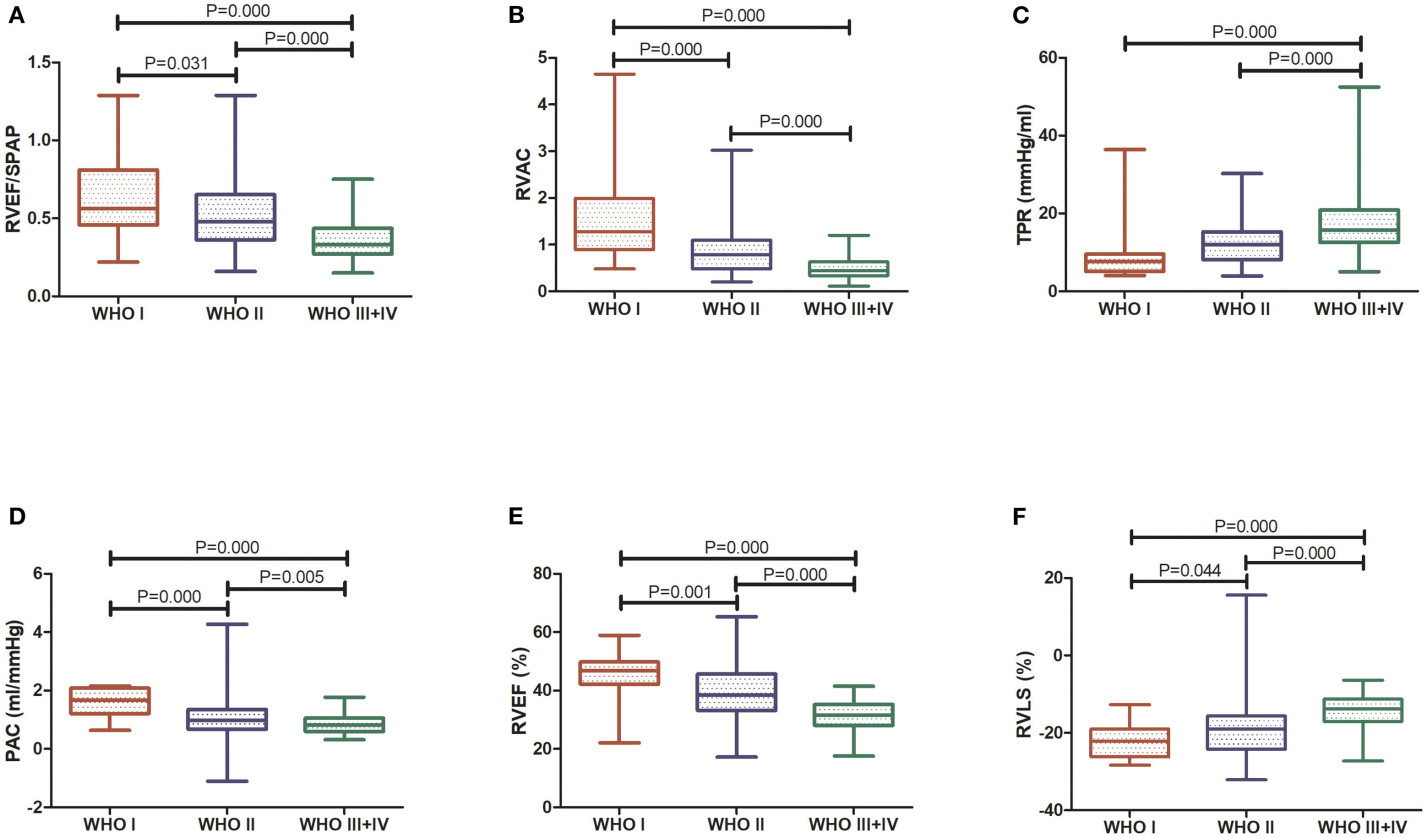
Comparison of RV function indices and RV coupling to pulmonary circulation among WHO-FC groups. **(A)** RVEF/PASP; **(B)** RVAC; **(C)** TPR; **(D)** PAC; **(E)** 3D RVEF; and **(F)** RVLS.

The RVAC and PAC were found to be lower and the TPR was higher in patients with precapillary PH with worsening WHO-FC stage. RV-PA coupling, as assessed by the RVEF/PASP ratio, was found to be lower in patients with precapillary PH with worsening WHO-FC, showing a decline across the WHO-FC stages consistent with worsening RV systolic function accounting for RV afterload. The results in Table 2 and Figure 2 show that patients with WHO-FC III+IV PH had a significantly lower RVEF/PASP ratio than patients with WHO-FC I and II PH (both *P* < 0.001), and the difference in the RVEF/PASP ratio was significant between patients with WHO-FC I and WHO-FC II (*P* < 0.031). RVAC and PAC also differed significantly among the WHO-FCs (*P* < 0.001). The TPR was found to be higher for WHO-FC III+IV than for WHO-FC I and II (*P* = 0.002 and *P* = 0.006, respectively), and no significant difference in TPR was observed between the WHO-FC I and II groups (*P* = 0.058).

Table 3 shows the correlations identified between the RVEF/PASP and TAPSE/PASP ratios and RV contractile function and its coupling to pulmonary circulation indices. Significant correlation was observed between the RVEF/PASP ratio and RVLS, and RVAC had a stronger correlation with the RVEF/PASP ratio than other indices (*r* = 0.701; *P* < 0.001). The correlations between the RVEF/PASP and TAPSE/PASP ratios and RV contractile function and its coupling to pulmonary circulation indices were similar, with no statistically significant differences.

**Table 3:** Correlations of RVEF/PASP and TAPSE/PASP ratios with RV contractile function and RV-PA coupling.

**Variable**	**Correlation with RVEF/PASP**				**Correlation with TAPSE/PASP**			
	**Pearson *r***	***P*-value**	**Spearman *r***	***P*-value**	**Pearson *r***	***P*-value**	**Spearman *r***	***P*-value**
RVLS	−0.565	<0.001	−0.658	<0.001	−0.594	<0.001	−0.635	<0.001
RVAC	0.622	<0.001	0.701	<0.001	0.588	<0.001	0.671	<0.001
TPR	−0.524	<0.001	−0.611	<0.001	−0.450	<0.001	−0.536	<0.001
PAC	0.472	<0.001	0.485	<0.001	0.513	<0.001	0.552	<0.001

### Association of RV Function and RV-PA Coupling With Adverse Outcomes

**Figure 3:**
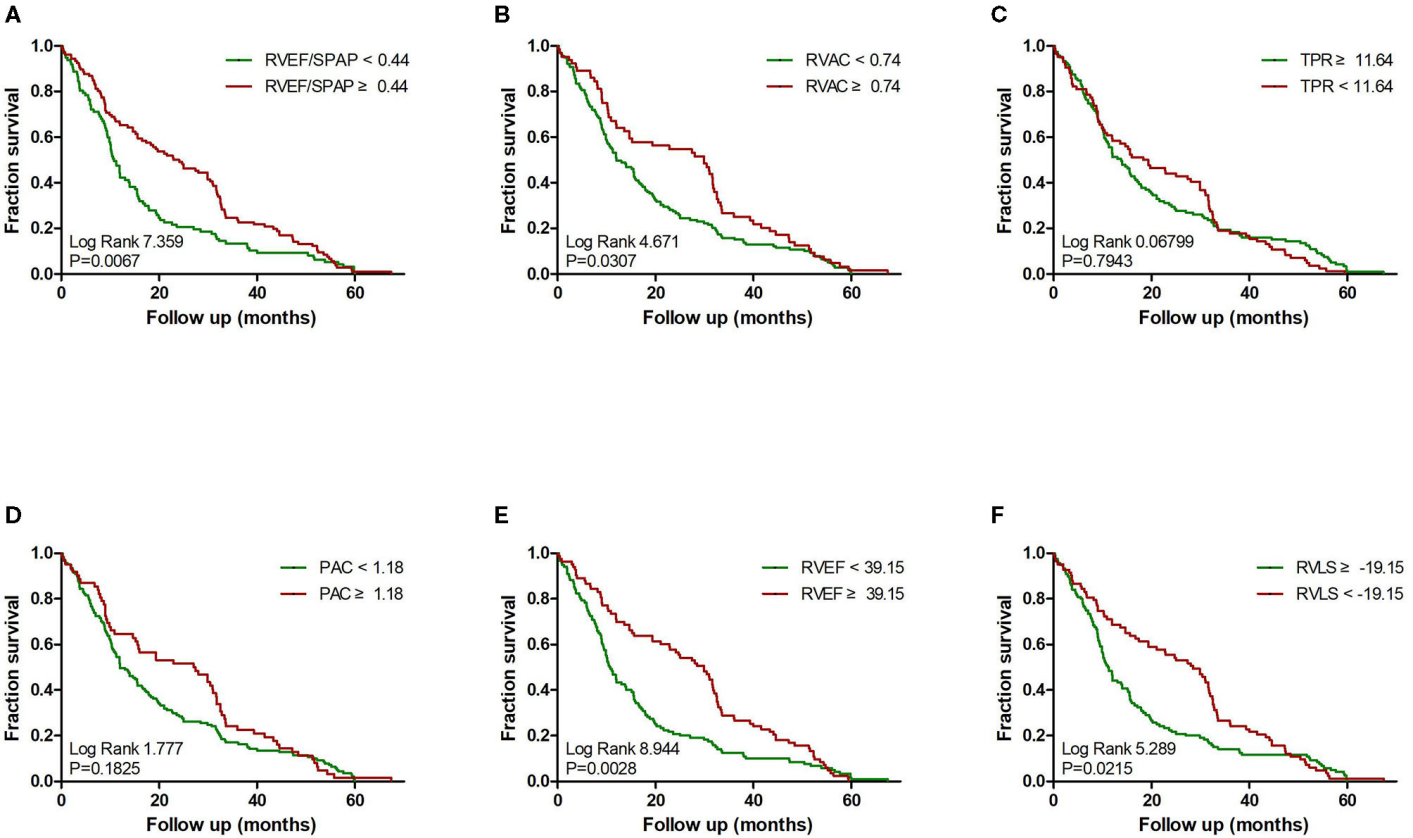
Impact of RV function indices and RV coupling to pulmonary circulation on clinical failure in precapillary PH. Kaplan–Meier survival analysis for precapillary PH patients with: **(A)** RVEF/PASP <0.44 and ≥0.44; **(B)** RVAC <0.74 and ≥0.74; **(C)** TPR <11.64 mm Hg/ml and ≥11.64 mm Hg/ml; **(D)** PAC <1.18 ml/mm Hg and ≥1.18 ml/mm Hg; **(E)** 3D RVEF <39.15% and ≥39.15%; and **(F)** RVLS < -19.15% and ≥-19.15%.

Multivariate Cox proportional-hazard analysis showed that a lower 3D RVEF and worse RVLS were strong predictors of adverse clinical events (HR, 0.907; 95% CI, 0.857–0.959; *P* = 0.001 and HR, 1.047; 95% CI, 1.001–1.096; *P* = 0.046, respectively). Another multivariate predictor of clinical worsening was TPR (HR, 0.954; 95%CI, 0.915–0.995; *P* = 0.027) after adjustment for age, sex and BSA. The RVEF/PASP ratio did not show significant predictive value (Table 4). Kaplan–Meier survival curves demonstrated the relationship between RV function and RV-PA coupling indices and clinical worsening (Figure 3).

**Table 4:** Cox regression analysis of factors predictive of adverse clinical outcomes in precapillary PH patients after adjustment for age, sex, and BSA.

	**Univariate analysis**		**Multivariable analysis**	
**Variables**	**Adjusted HR** ** (95% CI)**	***P*-value**	**Adjusted HR** ** (95% CI)**	***P*-value**
6-min walk distance	1.001 (0.998–1.005)	0.467		
WHO functional class^*^	2.389 (0.755–7.557)	0.255		
**RV-PA coupling**				
RVEF	0.896 (0.842–0.954)	**0.001**	0.907 (0.857–0.959)	**0.001**
RVLS	1.051 (1.005–1.101)	**0.031**	1.047 (1.001–1.096)	**0.046**
RVEF/PASP	2.688 (0.182–37.321)	0.472	2.026 (0.162–25.363)	0.584
TAPSE/PASP	2.483 (0.165–39.792)	0.511	2.389 (0.155–27.557)	0.561
RVAC	0.687 (0.272–1.732)	0.426	0.661 (0.301–1.449)	0.301
TPR	0.858 (0.779–0.945)	**0.002**	0.954 (0.915–0.995)	**0.027**
PAC	0.421 (0.213–0.833)	**0.013**	0.576 (0.311–1.065)	0.079
**Hemodynamics data**				
Cardiac index	0.981 (0.519–1.853)	0.953		
Pulmonary vascular resistance	1.001 (1.000–1.002)	0.059		

*HR, hazard ratio per unit increase in the parameter measured*;

* *HR per category in the measured parameter. Bold values indicate statistical significance of the tested parameters*.

**Figure 4:**
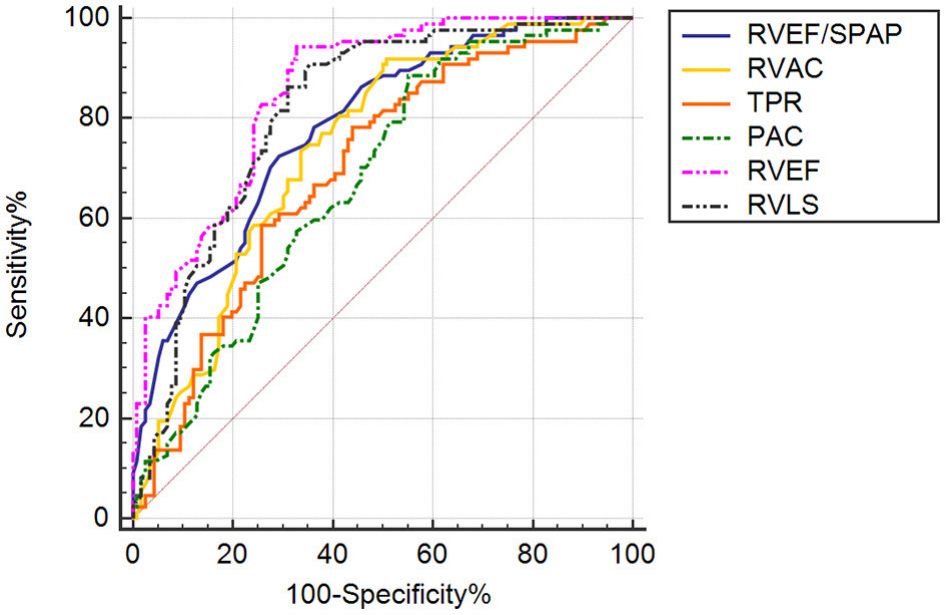
ROC curve analysis of RV function indices and RV coupling to pulmonary circulation in patients with precapillary PH. AUC, area under the curve. ROC curve analysis demonstrating the abilities of RVEF/PASP, RVAC, TPR, PAC, 3D RVEF, and RVLS to predictive adverse clinical outcomes in PH patients.

The calculated optimal cutoff values for the most significant parameters are presented in Table 5. Precapillary-PH with RV-PA uncoupling as expressed by a RVEF/PASP ratio <0.44 predicted adverse clinical outcomes, with a sensitivity of 70.7% and specificity of 72.4%. Receiver operating characteristic (ROC) curve analysis confirmed that RVEF and the strongest predictive power among the tested parameters [area under the curve (AUC) = 0.855; 95% CI, 0.805–0.905] and identified a RVEF of 39.1% as the best cutoff value (Figure 4), with 67.2% sensitivity and 94.3% specificity. RVAC, TPR, and PAC had varying degrees of predictive value (*P* < 0.001), with optimal cutoff values of 0.74, 11.64, and 1.18, respectively.

**Table 5:** ROC curve analysis determining the optimal cutoff values of tested parameters.

**Variables**	**Optimal** ** cutoff**	**AUC**	**95% CI**	***P***	**Sensitivity** ** (%)**	**Specificity** ** (%)**
RVEF (%)	39.15	0.855	0.805–0.905	<0.001	67.2	94.3
RVLS (%)	−19.15	0.816	0.757–0.875	<0.001	90.8	64.7
RVEF/PASP	0.44	0.783	0.721–0.845	<0.001	70.7	72.4
RVAC	0.74	0.743	0.676–0.810	<0.001	66.4	74.7
TPR	11.64	0.698	0.626–0.770	<0.001	78.2	56
PAC	1.18	0.676	0.603–0.749	<0.001	44.8	88.5

*AUC, Area under the curve; NPV, negative predictive value; PPV, positive predictive value*.

### Reproducibility Analysis

We next assessed the manual correction reproducibility of RVEDV, RVESV, and RVEF using Bland-Altman analysis. The intra-observer variability for RVEDV was −3.04 ± 5.39 (95% CI, −13.61–7.53), and the inter-observer variability was −3.65 ± 6.36 (95% CI, −16.11–8.81). The intra-observer variability for RVESV was −2.21 ± 3.58 (95% CI, −9.22–4.80), and the inter-observer variability was −2.20 ± 4.25 (95% CI, −10.54–6.13). The intra-observer variability for RVEF was 0.14 ± 0.83 (95% CI, −1.49–1.77), and the inter-observer variability was −0.02 ± 1.06 (95% CI, −2.10–2.06).

## Discussion

In the present study, we quantified RV function and RV-PA coupling in precapillary PH patients, and our results provide insights based on 3D echocardiographic non-invasive measurement into potential predictors of RV dysfunction and RV coupling to the pulmonary circulation as well as their associations with patient prognosis. First, we observed that RV systolic function declined progressively within advancing WHO-FC, and 3D RVEF and RVLS demonstrated strong independent predictive value. Second, precapillary-PH in our study with a RVEF/PASP ratio <0.44 was associated with adverse clinical outcomes. Third, PAC decreased and TPR increased with advancing WHO-FC, with TPR showing the best independent predictive value.

Chronic RV failure occurs in four distinct stages due to alterations in preload; changes in RV mechanics, and/or contractility; or increases in afterload (6). RV maladaptive remodeling takes place with excessive hypertrophy, progressive RV dilatation, and dyssynchrony. Determination of RV dimensions and volumes is essential to the diagnosis and prognostication of right heart failure (19). It is generally known that the RV is particularly afterload sensitive, such that RVEF is inversely associated with PVR in PH conditions. In our study, RV function deteriorated (lower RVEF and worse RVLS) with advancing WHO-FC in patients with precapillary PH. 3D-RVEF, and as a sensitive index, had independent predictive value for poor prognosis. In this study, 3D RVEF measurements were acquired using semi-automatic software, which offers a rapid and simple technique with high repeatability.

We were able to evaluate RV-PA coupling performance relative to the RVEF/PASP ratio derived from 3DE. From the perspective of physiology and clinical treatment, it is very important and necessary to analyze the cardiopulmonary unit as a system. RV adaptability to PH depends on the ability of the RV to respond to increased afterload and increase its contractility. RVAC refers to the relationship between ventricular contractility and afterload, and the most objective index is the ratio of ventricular elasticity to arterial elasticity (20). A 2018 ARIC study of 1,004 elderly participants also showed that a lower RVEF and lower RVEF/PASP ratio are linearly associated with an increased risk of incident heart failure or death, suggesting an important and underrecognized role of RV dysfunction in the progression to heart failure. That study also suggested the potential utility of 3DE for accurate assessment of RV performance (21–23).

Compared to TAPSE as a single measure, TAPSE/PASP shows more pronounced differences in pediatric PH with different NYHA FC/modified ROSS score values (24). Tello et al. validated TAPSE/PASP as a reliable parameter for RV-PA coupling assessment in patients with severe idiopathic and thromboembolic PH. They recommended the echocardiographic TAPSE/PASP ratio as a straightforward surrogate for the invasively measured Ees/Ea to assess RV-arterial coupling (12). In our study, the correlations between the RVEF/PASP and TAPSE/PASP ratios and RV contractile function and its coupling to pulmonary circulation indices were similar, with no statistically significant differences. TAPSE is afterload-dependent, plane-dependent, and falsely increased in the presence of severe tricuspid regurgitation, although this parameter is very easily accessible. It is well-established that 3DE allows accurate and repeatable measurements of RV size and function. Most recently, the application of artificial intelligence methods, including machine learning algorithm techniques, have been developed for automatic detection of RV intima boundaries. 3DE uniquely allows for the acquisition of a 3D volumetric dataset from which RV volumes and EF can be quantified without geometric assumptions (25, 26). The right ventricle will continue to be the focus of intensive research as this topic challenges both clinicians and scientists. Computational methods will potentially contribute to improved diagnosis and risk stratification algorithms for right heart failure and contribute to automation of image analysis (27).

PAC was considered as an increase in blood volume in the arterial system, which produces a unit increase in arterial transmural pressure. We demonstrated that PAC decreased and TPR increased with advancing WHO-FC in the present study. PAC depends on the volume of the reservoir and on the elastic properties of the PA, as determined by the wall content and mPAP (28). A significant proportion of the RV afterload is determined by the elastic properties of the PA. However, a significant proportion of RV energy is spent generating pulsations, and such requirements are imposed by the elastic properties of the PA (29). The preoperative PAC can be an independent predictor of adverse postoperative hemodynamics (30). PAC was previously shown to be an important prognostic factor in PAH, and Guigui et al. showed that the relationship between PAC and PVR remained stable at mid-term follow-up, suggesting that compliance is an important disease marker beyond the early stages (31).

### Study Limitations

Limitations of our study include its single-center design and limited sample size. Further multicenter studies are needed to confirm these findings. Patients with precapillary PH had different types of PH, with CTEPH being most common (95/203, 46.8%). There were differences in treatment options between patients, which may have biased the patients' prognosis. RV Ees was obtained by RHC and was determined using the single-beat method (32). We calculated RV-PA coupling using echocardiographic 3D volumes and hemodynamic parameters of the right cardiac catheter, and subsequent studies require further validation of our results through pressure-volume catheterization. *P*_max_ was not calculated from measurements in the RHC procedure, and mPAP was used as a surrogate for RV ESP. Recent studies have shown that using mPAP as an alternative to RVESP may not be the best approach in RV–arterial coupling in severe PAH and have suggested that evaluating RVESP is best computed with modified equations (33).

## Conclusion

A progressive increase in afterload causes RV dysfunction in patients with precapillary PH, and 3D RVEF and RVLS demonstrated strong independent predictive value. RV-PA coupling, assessed according to the RVEF/PASP ratio, declined with advancing WHO-FC, consistent with worse RV systolic function accounting for RV afterload. Precapillary-PH with RV-PA uncoupling as expressed by a RVEF/PASP ratio <0.44 was associated with adverse clinical outcomes. TPR increased with advancing WHO-FC stage and showed independent predictive value.

## Data Availability Statement

The raw data supporting the conclusions of this article will be made available by the authors, without undue reservation.

## Ethics Statement

The studies involving human participants were reviewed and approved by Ethics Committee of Beijing Chao-yang Hospital. The patients/participants provided their written informed consent to participate in this study.

## Author Contributions

YL designed the study and drafted and wrote the manuscript. All clinical characteristics and laboratory data at baseline were obtained from standardized clinical data by YY and JG. RHC were performed JW and QH. YL, DG, XZ, HH, and ZJ collected and analyzed the data. DG and SY drafted and wrote the manuscript. YL, XL, and YY revised the manuscript critically for intellectual content. All authors gave intellectual input to the study and approved the final version of the manuscript.

## Conflict of Interest

SY was employed by the company Philips (China) Investment Co. Ltd. The remaining authors declare that the research was conducted in the absence of any commercial or financial relationships that could be construed as a potential conflict of interest.

## Supplementary Material

Supplemental Figure 1 Example of machine learning-based 3D RVEF analysis using QLAB 13.0 software. End-diastolic and systolic verification and editing of endocardial RV borders, from which RVEDV, RVESV, and RVEF were obtained.

## Abbreviations

AUC: area under the curve
BSA: body surface area
CI: Cardiac index
FAC: fractional area change
ICCs: intraclass correlation coefficients
PAP: pulmonary artery pressure
ROC: receiver operating characteristic
RV: right ventricular
RVEF: right ventricular ejection fraction
RVLS: RV longitudinal strain
PASP: PA systolic pressure
PAC: pulmonary arterial compliance
TPR: total pulmonary resistance
WHO-FC: World Health Organization functional class
RVAC: RV arterial coupling
PAWP: pulmonary wedge pressure
PCWP: pulmonary capillary wedge pressure
RHC: right heart catheterization
PA: pulmonary arterial
SD: standard deviation
Ea: effective arterial elastance
Ees: end-systolic elastance
MRI: magnetic resonance imaging
TAPSE: tricuspid annular plane systolic excursion
NT-proBNP: N-terminal pro-brain natriuretic peptide
EDV: end-diastolic volume
ESV: end-systolic volume
SVI: stroke volume index
RIMP: RV index of myocardial performance
6MWD: 6-minute walk distance.
