# Supplementary figures and images for: Right Ventricular Function and Its Coupling With Pulmonary Circulation in Precapillary Pulmonary Hypertension: A Three-Dimensional Echocardiographic Study
Source: Front Cardiovasc Med. 2021 Jul 2;8:690606. doi: 10.3389/fcvm.2021.690606 (PMC8282926; doi:10.3389/fcvm.2021.690606)

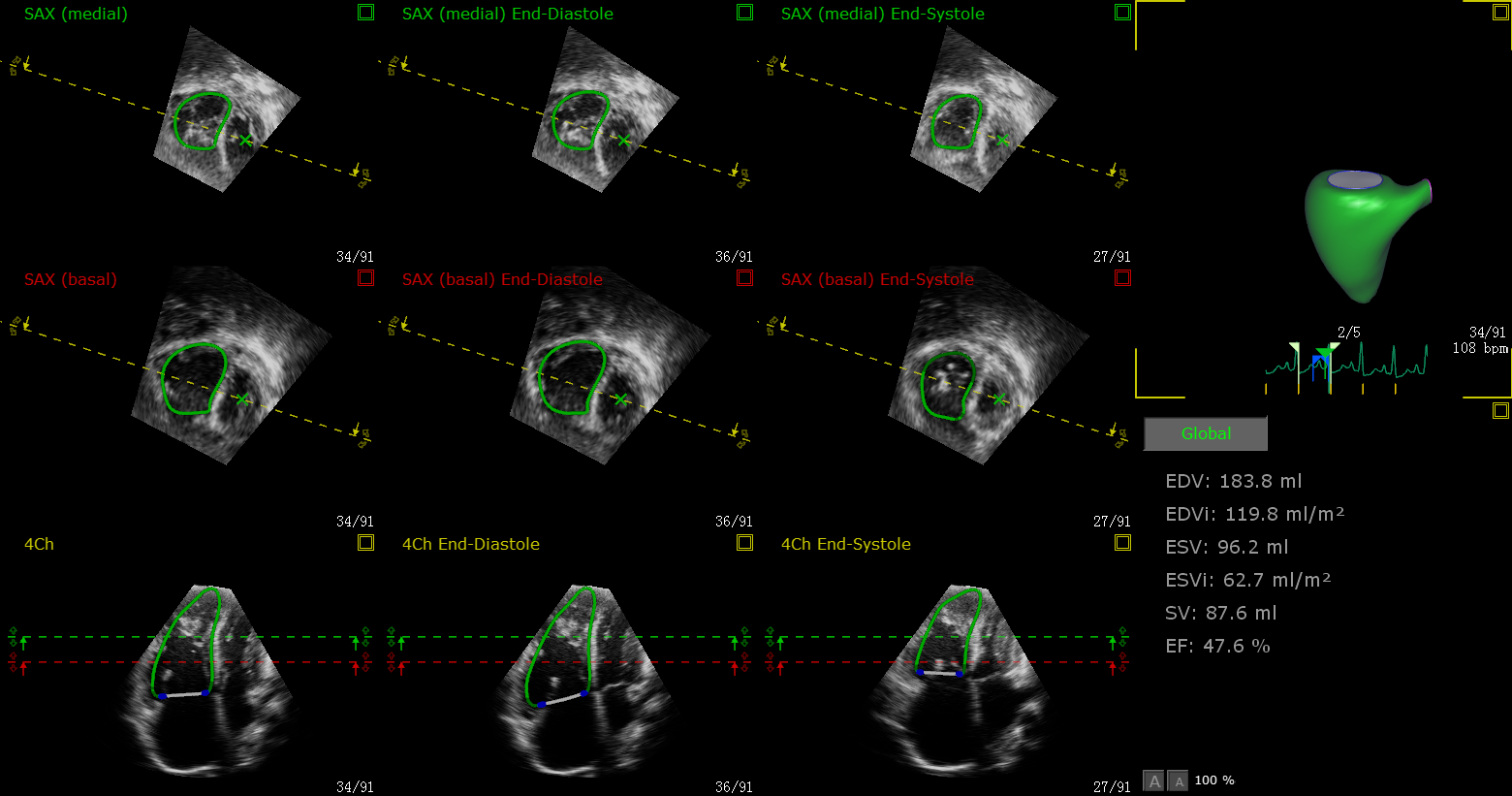

Supplement: Supplemental Figure 1 — Example of machine learning-based 3D RVEF analysis using QLAB 13.0 software. End-diastolic and systolic verification and editing of endocardial RV borders, from which RVEDV, RVESV, and RVEF were obtained. [file Image_1.TIF]
